# Fast Nonlinear Predictive Control Using Classical and Parallel Wiener Models: A Comparison for a Neutralization Reactor Process

**DOI:** 10.3390/s23239539

**Published:** 2023-11-30

**Authors:** Robert Nebeluk, Maciej Ławryńczuk

**Affiliations:** Institute of Control and Computation Engineering, Faculty of Electronics and Information Technology, Warsaw University of Technology, ul. Nowowiejska 15/19, 00-665 Warsaw, Poland; maciej.lawrynczuk@pw.edu.pl

**Keywords:** model predictive control, wiener models, neutralization reactor

## Abstract

The Wiener model, composed of a linear dynamical block and a nonlinear static one connected in series, is frequently used for prediction in Model Predictive Control (MPC) algorithms. The parallel structure is an extension of the classical Wiener model; it is expected to offer better modeling accuracy and increase the MPC control quality. This work discusses the benefits of using the parallel Wiener model in MPC. It has three objectives. Firstly, it describes a fast MPC algorithm in which parallel Wiener models are used for online prediction. In the presented approach, sophisticated trajectory linearization is performed online, which leads to computationally fast quadratic optimization. The second objective of this work is to study the influence of the model structure on modeling accuracy. The well-known neutralization benchmark process is considered. It is shown that the parallel Wiener models in the open-loop mode generate significantly fewer errors than the classical structure. This work’s third objective is to validate the efficiency of parallel Wiener models in closed-loop MPC. For the neutralization process, it is demonstrated that parallel models demonstrate better control quality using various indicators, but the difference between the classical and parallel models is not significant.

## 1. Introduction

Model Predictive Control (MPC) refers to an advanced control strategy in which a dynamical model of the considered process is utilized online to predict the future process state and an optimization procedure finds the best possible control action to minimize the predefined control quality index [1]. MPC algorithms have been used for years in process control; typical applications include chemical reactors [2], olefin metathesis processes [3], distillation towers [4] and power plants [5]. Nowadays, as a result of the availability of fast and relatively cheap hardware platforms necessary to carry out all online calculations, MPC algorithms are used in smart buildings [6] and several embedded systems; example applications include autonomous ground vehicle [7], autonomous driving vehicle [8], planning vehicle-parking trajectories for vertical parking spaces [9] and quadrotors [10,11]. Finally, MPC algorithms may control distributed parameter systems [12].

Two factors are essential for good control quality: precise online measurements provided by sensors and an accurate model of the controlled process. MPC algorithms utilize measurements of the process output variables (and state variables, in some cases). Significant measurement errors combined with an imprecise model result in poor predictions and, in consequence, unsatisfactory control performance. The importance of precise measurements is stressed in [13] where a wind disturbance preview is incorporated with an MPC algorithm to improve the resistance of Unmanned Aerial Vehicles during operation to wind gusts. However, observers are designed to solve this critical problem in many scenarios if there are not enough process data available. By estimating the values in such a way, a better control quality is achievable. A state observer scheme is proposed in [14] for uninterruptible power supply applications and it is compared to classical approaches like with Kalman filters. The study presented in [15] shows an approach to deal with ocean environment disturbances by designing a nonlinear disturbance observer for unmanned surface vehicles to obtain safe and effective motion control performance. Lastly, in work [7], a Dual-Rate Extended Kalman filter is designed to obtain fast vehicle state estimation in the problem of real-time lane-keeping control for autonomous ground vehicles.

In this work, we study the impact of model structure and accuracy on the possible control performance. Although the general idea of MPC does not limit the model structure used for online prediction, the cascade Wiener model is frequently used [16]. The Wiener model consists of a linear dynamical block connected in series with a nonlinear static block. A great advantage of the Wiener model is the fact that it can efficiently approximate the properties of different processes using a limited number of parameters. Let us name a few examples reported in the literature: distillation columns [17], chemical reactors [18,19,20], gasifiers [21], chromato-graphic separation processes [22], fuel cells [23,24], photovoltaic cells [25], the relaxation processes during anesthesia [26], the arterial pulse transmission phenomena [27]. Additionally, due to the specialized structure of the Wiener model, we can derive a set of computationally efficient MPC algorithms in which fast quadratic optimization is used rather than complicated nonlinear programming [16].

Typically, the classical Wiener model structure is used in MPC [16], i.e., the model consists of one linear dynamical block and one nonlinear static block. A natural extension of the rudimentary Wiener structure uses a few classical sub-models connected in parallel. Such a model structure and identification issues are described in [28,29], while identification starting from linearized models is considered in [30]. The motivation to use the parallel structure is the following: the parallel model should be capable of generating better accuracy than the classical one. As a result, the parallel model is likely to offer better control quality when used in MPC compared to the classical model structure. Of course, this may be true for some processes, while for other ones, the classical structure may be sufficient.

This work has the following three objectives:

1. The first objective is to extend previous research in computationally efficient MPC algorithms in which Wiener models are used for prediction [16]. Namely, the goal is to detail a fast MPC method in which a linear approximation of the process predicted trajectory is successively obtained online using parallel Wiener models. As a result, the derived MPC algorithm requires relatively simple and fast quadratic optimization rather than a nonlinear approach.
2. The second objective of this work is to study the influence of the model structure on modeling accuracy. We compare the accuracy of the classical Wiener structure and that of the parallel Wiener models. In the latter case, the impact of the number of sub-models and the complexity of the nonlinear block are thoroughly evaluated. To the best of the authors’ knowledge, a fair comparison between the classical and parallel Wiener models has not yet been presented in the literature.
3. The third objective of this work is to compare the efficiency of classical and parallel Wiener models in MPC. The problem is really important. Although more sophisticated models are likely to produce much better modeling accuracy in an open loop, the advantages of using complex models may be insignificant in MPC. Multi-criteria control quality assessment is used to demonstrate the impact of model structure.

The well-known neutralization benchmark process [19] is considered to verify the advantages of parallel Wiener models used in the open loop and MPC. Precise modeling and control of the neutralization benchmark process is essential in different areas, i.e., in chemical engineering, biotechnology and waste-water treatment industries [31]. Moreover, it is often utilized as a benchmark to assess the efficiency of new model structures and control algorithms, e.g., [16,32,33,34,35,36,37,38].

This work is structured as follows. Section 2 defines the structure of the classical Wiener model and its parallel variant, Section 3 derives and discusses the implementation of the fast MPC algorithm for the parallel Wiener model, Section 4 thoroughly discusses simulation results and Section 5 summarizes the whole work.

## 2. Classical and Parallel Wiener Models

**Figure 1:**
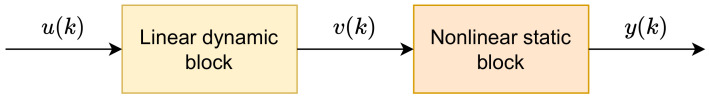
Classical Wiener model structure.

Let us start with the definition of the classical Wiener model [16,39]. In this work, we study Single-Input Single-Output (SISO) systems, i.e., we consider processes with one input and one output. The process input, which is also the manipulated variable in MPC, is denoted by *u*. The process output, which is the controlled variable in MPC, is denoted by *y*. Figure 1 shows the classical Wiener model consisting of a linear dynamic block and a nonlinear static block connected in series. Let us describe the model using mathematical formulas. We use the discrete-time description; *k* denotes the current sampling instant (k=0,1,2,…). The output signal of the linear block is

(1) $$v\left(k\right)=\sum_{i=1}^{n_{B}}b_{i}u\left(k-i\right)-\sum_{j=1}^{n_{A}}a_{j}v\left(k-j\right),$$

where integer numbers A and B define the order of model dynamics while real numbers $a_{j}$ and $b_{j}$ stand for model coefficients. The output signal of the second block, which is also the output of the whole Wiener model, is a nonlinear static mapping

(2) y(k)=f(v(k)).

Because we use online linearization of the predicted trajectory in MPC, we limit our considerations to differentiable functions *f*. In order to obtain precise models, we use neural networks with two layers, known to be universal approximators. Hence, the second block of the model is defined by

(3) $$y\left(k\right)=f\left(v\left(k\right)\right)=w_{0}^{2}+\sum_{i=1}^{K}w_{i}^{2}\varphi \left[w_{i,0}^{1}+w_{i,1}^{1}\left(v\left(k\right)\right)\right].$$

The first (hidden) layer is nonlinear; it has *K* hidden neurons and φ stands for the activation function, e.g., φ=tanh. The second layer of the network is linear. The weights of the first layer are denoted by $w_{i,0}^{1}$ and $w_{i,1}^{1}$, while the parameters of the second layer are $w_{0}^{2}$ and $w_{i}^{2}$.

**Figure 2:**
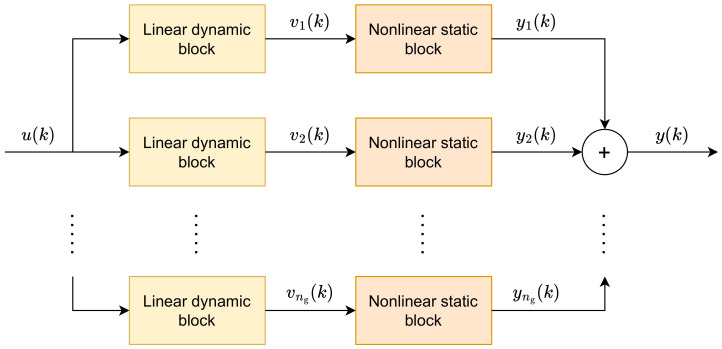
Parallel Wiener model structure.

The general structure of the parallel Wiener model [28,29,30] is depicted in Figure 2. The model consists of $n_{g}$ sub-models, also called branches, each of which has the classical Wiener structure. The model branches are connected in parallel; the outputs of the submodels are summarized. The outputs of the linear dynamic blocks are denoted by $v_{1}\left(k\right),\ldots ,v_{n_{g}}\left(k\right)$ while the outputs of the nonlinear static blocks are denoted by $y_{1}\left(k\right),\ldots ,y_{n_{g}}\left(k\right)$. Outputs of the consecutive linear blocks are calculated from the following formula

(4) $$v_{g}\left(k\right)=\sum_{i=1}^{n_{B}}b_{i,g}u\left(k-i\right)-\sum_{j=1}^{n_{A}}a_{j,g}v_{g}\left(k-j\right),$$

where $g=1,\ldots ,n_{g}$. The nonlinear blocks use neural networks with two layers and are described as follows

(5) $$y_{g}\left(k\right)=f_{g}\left(v_{g}\left(k\right)\right)=w_{0}^{2,g}+\sum_{i=1}^{K_{g}}w_{i}^{2,g}\varphi \left[w_{i,0}^{1,g}+w_{i,1}^{1,g}\left(v_{g}\left(k\right)\right)\right],$$

where *K* is the number of hidden neurons. The output of the whole model is calculated from Equation (5) from

(6) $$y\left(k\right)=\sum_{g=1}^{n_{g}}\left[w_{0}^{2,g}+\sum_{i=1}^{K_{g}}w_{i}^{2,g}\varphi \left[w_{i,0}^{1,g}+w_{i,1}^{1,g}\left(v_{g}\left(k\right)\right)\right]\right].$$

## 3. Predictive Control Using Classical and Parallel Wiener Models

### 3.1. Preliminaries

At each discrete sampling instant of MPC, i.e., k=0,1,2,…, the algorithm calculates the whole decision vector, which consists of increments of the manipulated variable signal for the current and future instants,

(7) $$▵u\left(k\right)=\left[\begin{array}{c} ▵u(k|k)\\ \vdots \\ ▵u(k+N_{u}-1|k) \end{array}\right],$$

where the number of the calculated increments is defined by the control horizon denoted by $N_{u}$. At the current sampling instant, only the first element of the calculated vector is applied to the process, and calculations are repeated at the following instants. Let us recall the rudimentary MPC optimization task, [1,16]

(8) $$\begin{array}{cc} \; & \min_{▵u(k)}\left\{J\left(k\right)=\sum_{p=1}^{N}{\left(y^{\mathrm{sp}}\left(k+p|k\right)-\hat{y}\left(k+p|k\right)\right)}^{2}+\lambda \sum_{p=0}^{N_{u}-1}{\left(▵u(k+p|k)\right)}^{2}\right\},\\ \; & \mathrm{subject}\;\mathrm{to}\\ \; & u^{\min}\leq u\left(k+p|k\right)\leq u^{\max},\;p=0,\ldots ,N_{u}-1,\\ \; & ▵u^{\min}\leq ▵u\left(k+p|k\right)\leq ▵u^{\max},\;p=0,\ldots ,N_{u}-1,\\ \; & y^{\min}\leq \hat{y}\left(k+p|k\right)\leq y^{\max},\;p=0,\ldots ,N-1. \end{array}$$

The objective of MPC is to find online the decision variable vector, ▵u(k), that minimizes the predefined cost function, J(k), and satisfies all constraints. As far as the cost function is concerned, we consider predicted control errors, defined as differences between the setpoint trajectory, $y^{\mathrm{sp}}\left(k+p|k\right)$, and the predicted trajectory, $\hat{y}\left(k+p|k\right)$, which is found from the process model. As many as *N* predicted control errors are considered; *N* is called the prediction horizon. The second part of the cost function minimizes unwanted significant changes in the manipulated variable; λ stands for the penalty coefficient. In this work, we consider classical MPC constraints, i.e., it is possible to consider limitations of the magnitude of the manipulated variable, the increments of that variable and the magnitude of the predicted value of the controlled variable.

### 3.2. Derivation of Fast MPC Algorithm

Let us note that as a result of model nonlinearity, predictions $\hat{y}\left(k+p|k\right)$ are nonlinear functions of the calculated MPC decision vector, ▵u(k). It means that the MPC optimization task (8) is nonlinear, and a nonlinear solver is necessary at each sampling instant. This work adopts the MPC Algorithm with Nonlinear Prediction and Linearization along with the Predicted Trajectory (MPC-NPLPT) derived in [16] for the classical Wiener model. The MPC algorithm discussed next requires that the dynamical model used for prediction can be linearized online. It is true when the neural Wiener models described in Section 2 are differentiable. This assumption is fulfilled when activation function φ used in the nonlinear hidden nodes of the models’ static blocks is differentiable. It is true for φ=tanh.

Let us define the predicted output trajectory vector

(9) $$\hat{y}\left(k\right)=\left[\begin{array}{c} \hat{y}\left(k+1|k\right)\\ \vdots \\ \hat{y}\left(k+N|k\right) \end{array}\right].$$

The idea behind the MPC-NPLPT algorithm is to use a linear approximation of the predicted trajectory with respect to the decision vector, ▵u(k). Trajectory linearization is performed along some predefined trajectory of the manipulated variable

(10) $$u^{\mathrm{traj}}\left(k\right)=\left[\begin{array}{c} u^{\mathrm{traj}}\left(k|k\right)\\ \vdots \\ u^{\mathrm{traj}}\left(k+N_{u}-1|k\right) \end{array}\right].$$

Using the process model, we determine the predicted trajectory of the controlled variable that corresponds to the assumed trajectory $u^{\mathrm{traj}}\left(k\right)$

(11) $${\hat{y}}^{\mathrm{traj}}\left(k\right)=\left[\begin{array}{c} {\hat{y}}^{\mathrm{traj}}\left(k+1|k\right)\\ \vdots \\ {\hat{y}}^{\mathrm{traj}}\left(k+N|k\right) \end{array}\right].$$

In order to analytically derive trajectory ${\hat{y}}^{\mathrm{traj}}\left(k\right)$ over the whole prediction horizon, we have first to use Equation (4) to express the outputs of the first block of the model explicitly predicted for sampling instant k+p at current instant *k*

(12) $$v_{g}^{\mathrm{traj}}\left(k+p|k\right)=\sum_{i=1}^{n_{B}}b_{i,g}u^{\mathrm{traj}}\left(k-i+p|k\right)-\sum_{j=1}^{n_{A}}a_{j,g}v_{g}^{\mathrm{traj}}\left(k-j+p|k\right).$$

Next, we use Equation (6) to express the outputs of the second block of the model, which is the model output. The predicted model output signal is

(13) $${\hat{y}}^{\mathrm{traj}}\left(k+p|k\right)=\sum_{g=1}^{n_{g}}\left[w_{0}^{2,g}+\sum_{i=1}^{K_{g}}w_{i}^{2,g}\varphi \left[w_{i,0}^{1,g}+w_{i,1}^{1,g}\left(v_{g}^{\mathrm{traj}}\left(k+p|k\right)\right)\right]\right]+d\left(k\right).$$

Because a model is never perfect, in prediction Rule (13), we supplement the model output by an estimated model error denoted by d(k). It is determined straightforwardly as a difference between real (measured) process output denoted by y(k) and model output

(14) $$d\left(k\right)=y\left(k\right)-\sum_{g=1}^{n_{g}}\left[w_{0}^{2,g}+\sum_{i=1}^{K_{g}}w_{i}^{2,g}\varphi \left[w_{i,0}^{1,g}+w_{i,1}^{1,g}\left(v_{g}\left(k\right)\right)\right]\right].$$

As thoroughly derived in [16], the linear approximation of the predicted trajectory of the process output is given by the following vector–matrix formula

(15) $$\hat{y}\left(k\right)=H\left(k\right)J▵u\left(k\right)+{\hat{y}}^{\mathrm{traj}}\left(k\right)+H\left(k\right)\left(u\left(k-1\right)-u^{\mathrm{traj}}\left(k\right)\right).$$

The matrix of partial derivatives of the predicted output trajectory with respect to the input trajectory is of dimensionality $N\times N_{u}$ and has the following structure

(16) $$H\left(k\right)=\frac{d{\hat{y}}^{\mathrm{traj}}\left(k\right)}{du^{\mathrm{traj}}\left(k\right)}=\left[\begin{array}{ccc} \frac{\partial {\hat{y}}^{\mathrm{traj}}\left(k+1|k\right)}{\partial u^{\mathrm{traj}}\left(k|k\right)} & \cdots & \frac{\partial {\hat{y}}^{\mathrm{traj}}\left(k+1|k\right)}{\partial u^{\mathrm{traj}}\left(k+N_{u}-1|k\right)}\\ \vdots & \ddots & \vdots \\ \frac{\partial {\hat{y}}^{\mathrm{traj}}\left(k+N|k\right)}{\partial u^{\mathrm{traj}}\left(k|k\right)} & \cdots & \frac{\partial {\hat{y}}^{\mathrm{traj}}\left(k+N|k\right)}{\partial u^{\mathrm{traj}}\left(k+N_{u}-1|k\right)} \end{array}\right].$$

Let us now analytically derive entries of the matrix H(k) for the parallel Wiener shown in Figure 2. The partial derivatives are calculated differentiating Equation (13), which yields

(17) $$\frac{\partial {\hat{y}}^{\mathrm{traj}}\left(k+p|k\right)}{\partial u^{\mathrm{traj}}\left(k+r|k\right)}=\sum_{g=1}^{n_{g}}\sum_{i=1}^{K_{g}}w_{i}^{2,g}\frac{d\varphi (c_{i,g}^{\mathrm{traj}}\left(k+p|k\right))}{dc_{i,g}^{\mathrm{traj}}\left(k+p|k\right)}\frac{\partial c_{i,g}^{\mathrm{traj}}\left(k+p|k\right)}{\partial u^{\mathrm{traj}}\left(k+r|k\right)},$$

where predicted input signals of the first layer of neural networks used in the nonlinear static block of the Wiener model are

(18) $$c_{i,g}^{\mathrm{traj}}\left(k+p|k\right)=w_{i,0}^{1,g}+w_{i,1}^{1,g}\left(v_{g}^{\mathrm{traj}}\left(k+p|k\right)\right).$$

If the hyperbolic tangent (tanh) function is used as the neural network activation function φ, we have

(19) $$\frac{d\varphi (c_{i,g}^{\mathrm{traj}}\left(k+p|k\right))}{dc_{i,g}^{\mathrm{traj}}\left(k+p|k\right)}=1-{\left(\varphi \left(c_{i,g}^{\mathrm{traj}}\left(k+p|k\right)\right)\right)}^{2}.$$

Combining Equations (17) and (19), we obtain the general formula to determine the entries of matrix H(k)

(20) $$\frac{\partial {\hat{y}}^{\mathrm{traj}}\left(k+p|k\right)}{\partial u^{\mathrm{traj}}\left(k+r|k\right)}=\sum_{g=1}^{n_{g}}\sum_{i=1}^{K_{g}}w_{i,1}^{1,g}w_{i}^{2,g}\left(1-{\left(\varphi \left(c_{i,g}^{\mathrm{traj}}\left(k+p|k\right)\right)\right)}^{2}\right)\frac{\partial v_{g}^{\mathrm{traj}}\left(k+p|k\right)}{\partial u^{\mathrm{traj}}\left(k+r|k\right)}.$$

Partial derivatives in the right-hand side of Equation (20) are also calculated analytically. For this purpose, we differentiate Equation (18). As far as the prediction for the first sampling instant of the prediction horizon is concerned, i.e., for sampling instant k+1, we obtain

(21) $$\begin{array}{c} \frac{\partial v_{g}^{\mathrm{traj}}\left(k+1|k\right)}{\partial u^{\mathrm{traj}}\left(k+r|k\right)}=\left\{\begin{array}{cc} b_{1,g} & \mathrm{for}r=0\\ 0 & \mathrm{for}r>0 \end{array}\right.. \end{array}$$

It results in

(22) $$\frac{\partial {\hat{y}}^{\mathrm{traj}}\left(k+1|k\right)}{\partial u^{\mathrm{traj}}\left(k+r|k\right)}=0\;\mathrm{for}\;\mathrm{all}\;r>0.$$

Similarly, for the prediction for the second sampling instant of the prediction horizon, i.e., for sampling instant k+2, we obtain

(23) $$\begin{array}{cc} \frac{\partial v_{g}^{\mathrm{traj}}\left(k+2|k\right)}{\partial u^{\mathrm{traj}}\left(k+r|k\right)} & =\sum_{i=1}^{n_{B}}b_{i,g}\frac{\partial u^{\mathrm{traj}}\left(k-i+2|k\right)}{\partial u(k+r|k)}-\sum_{j=1}^{n_{A}}a_{j,g}\frac{\partial v_{g}^{\mathrm{traj}}\left(k-j+2|k\right)}{\partial u(k+r|k)}. \end{array}$$

Since the prediction horizon is typically longer than the control horizon, we have

(24) $$\frac{\partial u^{\mathrm{traj}}\left(k-i+p|k\right)}{\partial u^{\mathrm{traj}}\left(k+r|k\right)}=\left\{\begin{array}{cc} 1 & \mathrm{for}(r=i\mathrm{and}r<p)\\ 0 & \mathrm{otherwise} \end{array}\right..$$

In general, for the prediction for sampling instant k+p, we obtain

(25) $$\begin{array}{cc} \frac{\partial v_{g}^{\mathrm{traj}}\left(k+p|k\right)}{\partial u^{\mathrm{traj}}\left(k+r|k\right)} & =\sum_{i=1}^{n_{B}}b_{i,g}\frac{\partial u^{\mathrm{traj}}\left(k-i+p|k\right)}{\partial u(k+r|k)}-\sum_{j=1}^{n_{A}}a_{j,g}\frac{\partial v_{g}^{\mathrm{traj}}\left(k-j+p|k\right)}{\partial u(k+r|k)}. \end{array}$$

Let us stress that partial derivatives $\frac{\partial v_{g}^{\mathrm{traj}}\left(k-j+p|k\right)}{\partial u(k+r|k)}$ necessary in Equation (25) are calculated recurrently. Namely, calculations are repeated for all combinations of p=1,…,N and $r=0,\ldots ,N_{u}-1$ to find all entries of matrix H(k).

The auxiliary matrix of dimensionality $N_{u}\times N_{u}$ used in Equation (15) has the following structure

(26) $$J=\left[\begin{array}{ccccc} 1 & 0 & 0 & \ldots & 0\\ 1 & 1 & 0 & \ldots & 0\\ \vdots & \vdots & \vdots & \ddots & \vdots \\ 1 & 1\; & 1 & \ldots & 1 \end{array}\right],$$

and the auxiliary vector of length $N_{u}$ is

(27) $$u\left(k-1\right)=\left[\begin{array}{c} u(k-1)\\ \vdots \\ u(k-1) \end{array}\right].$$

Using the linear approximation of the predicted output trajectory given by Equation (15), the general MPC optimization task (8) is transformed into the following quadratic optimization task

(28) $$\begin{array}{cc} \; & \min_{▵u(k)}\{J\left(k\right)=∥y^{\mathrm{sp}}\left(k\right)-H\left(k\right)J▵u\left(k\right)-{\hat{y}}^{\mathrm{traj}}\left(k\right)\\ \; & \;\;\;\;-H\left(k\right)\left(u\left(k-1\right)-u^{\mathrm{traj}}\left(k\right)\right){∥}^{2}+{∥▵u(k)∥}_{\Lambda }^{2}\},\\ \; & \mathrm{subject}\;\mathrm{to}\\ \; & u^{\min}\leq J▵u\left(k\right)+u\left(k-1\right)\leq u^{\max},\\ \; & ▵u^{\min}\leq ▵u\left(k\right)\leq ▵u^{\max},\\ \; & y^{\min}\leq H\left(k\right)J▵u\left(k\right)+{\hat{y}}^{\mathrm{traj}}\left(k\right)+H\left(k\right)\left(u\left(k-1\right)-u^{\mathrm{traj}}\left(k\right)\right)\leq y^{\max}. \end{array}$$

The constraints are expressed using the following vectors of length $N_{u}$

(29) $$u^{\min}=\left[\begin{array}{c} u^{\min}\\ \vdots \\ u^{\min} \end{array}\right],\;u^{\max}=\left[\begin{array}{c} u^{\max}\\ \vdots \\ u^{\max} \end{array}\right],\;▵u^{\min}=\left[\begin{array}{c} ▵u^{\min}\\ \vdots \\ ▵u^{\min} \end{array}\right],\;▵u^{\max}=\left[\begin{array}{c} ▵u^{\max}\\ \vdots \\ ▵u^{\max} \end{array}\right]$$

and the vectors of length *N*

(30) $$y^{\min}=\left[\begin{array}{c} y^{\min}\\ \vdots \\ y^{\min} \end{array}\right],\;y^{\max}=\left[\begin{array}{c} y^{\max}\\ \vdots \\ y^{\max} \end{array}\right].$$

The MPC-NPLPT algorithm repeats online trajectory linearization and quadratic optimization a few times at each sampling instant. Namely, the future input trajectory along which linearization is determined, i.e., $u^{\mathrm{traj}}\left(k\right)$ (Equation (10)), is initially set as the “tail” of the optimal control sequence found at the previous sampling instant, i.e., without its first element, ▵u(k|k). Quadratic programming task (28) is then solved. If the controlled variable of the process is close to a required setpoint, the first element of the optimized solution vector is applied to the process. If this condition is not fulfilled, the calculated decision vector is used to form trajectory $u^{\mathrm{traj}}\left(k\right)$; linearization is performed, followed by solving the quadratic optimization task. A few such repetitions may be used at each sampling instant. In practice, five repetitions are sufficient [16].

### 3.3. Classical Formulation of the MPC Quadratic Optimization Task

Let us consider the classical formulation of the quadratic optimization task

(31) $$\begin{array}{cc} \; & \min_{x(k)}\left\{0.5x^{T}\left(k\right)H_{\mathrm{QP}}\left(k\right)x\left(k\right)+f_{\mathrm{QP}}^{T}\left(k\right)x\left(k\right)\right\},\\ \; & \mathrm{subject}\;\mathrm{to}\\ \; & A(k)x(k)\leq B(k),\\ \; & \mathrm{LB}\leq x(k)\leq \mathrm{UB}, \end{array}$$

where x(k)=▵u(k). From Equation (28), we derive the time-varying linear inequality constraints

(32) $$A\left(k\right)=\left[\begin{array}{c} -J\\ J\\ -H(k)J\\ H(k)J \end{array}\right],\;B\left(k\right)=\left[\begin{array}{c} -u^{\min}+u\left(k-1\right)\\ u^{\max}-u\left(k-1\right)\\ -y^{\min}+{\hat{y}}^{\mathrm{traj}}\left(k\right)+H\left(k\right)\left(u\left(k-1\right)-u^{\mathrm{traj}}\left(k\right)\right)\\ y^{\max}-{\hat{y}}^{\mathrm{traj}}\left(k\right)-H\left(k\right)\left(u\left(k-1\right)-u^{\mathrm{traj}}\left(k\right)\right) \end{array}\right]$$

while constant bounds are specified by

(33) $$\mathrm{LB}=▵u^{\min},\;\mathrm{UB}=▵u^{\max}.$$

Matrix $H_{\mathrm{QP}}\left(k\right)$ is the second-order derivative of the cost function, J(k), with respect to the decision variables, ▵u(k). The first-order derivative is

(34) $$\begin{array}{cc} \frac{dJ(k)}{d▵u(k)}= & -2J^{T}H^{T}\left(k\right)\left(y^{\mathrm{sp}}\left(k\right)-H\left(k\right)J▵u\left(k\right)-{\hat{y}}^{\mathrm{traj}}\left(k\right)-H\left(k\right)\left(u\left(k-1\right)-u^{\mathrm{traj}}\left(k\right)\right)\right)\\ \; & +2\Lambda ▵u(k)\\ \; & =2(J^{T}H^{T}\left(k\right)H\left(k\right)J+\Lambda )▵u\left(k\right)\\ \; & \;-2J^{T}H^{T}\left(k\right)\left(y^{\mathrm{sp}}\left(k\right)-{\hat{y}}^{\mathrm{traj}}\left(k\right)-H\left(k\right)\left(u\left(k-1\right)-u^{\mathrm{traj}}\left(k\right)\right)\right), \end{array}$$

while the second-order derivative becomes

(35) $$H_{\mathrm{QP}}\left(k\right)=\frac{d^{2}J\left(k\right)}{d{\left(▵u\left(k\right)\right)}^{2}}=2\left(J^{T}H^{T}\left(k\right)H\left(k\right)J+\Lambda \right).$$

Vector $f_{\mathrm{QP}}\left(k\right)$ is defined by the part of the first-order derivative (34) which is independent of vector ▵u(k). We obtain

(36) $$f_{\mathrm{QP}}\left(k\right)=-2J^{T}H^{T}\left(k\right)\left(y^{\mathrm{sp}}\left(k\right)-{\hat{y}}^{\mathrm{traj}}\left(k\right)-H\left(k\right)\left(u\left(k-1\right)-u^{\mathrm{traj}}\left(k\right)\right)\right).$$

## 4. Simulations

### 4.1. Neutralization Process Description

In this work, we consider a neutralization reactor benchmark process to validate and compare the efficiency of classical and parallel Wiener models for open-loop modeling purposes and in closed-loop MPC control. The fundamental model of this benchmark process is described in detail in [19]. It consists of two differential equations and one algebraic equation. The full model formulation is as follows

(37) $$\begin{array}{cc} \frac{dW_{a}\left(t\right)}{dt} & =\frac{q_{1}\left(t\right)\left(W_{a_{1}}-W_{a}\left(t\right)\right)}{V}+\frac{q_{2}\left(W_{a_{2}}-W_{a}\left(t\right)\right)}{V}+\frac{q_{3}\left(W_{a_{3}}-W_{a}\left(t\right)\right)}{V}, \end{array}$$

(38) $$\begin{array}{cc} \frac{dW_{b}\left(t\right)}{dt} & =\frac{q_{1}\left(t\right)\left(W_{b_{1}}-W_{b}\left(t\right)\right)}{V}+\frac{q_{2}\left(W_{b_{2}}-W_{b}\left(t\right)\right)}{V}+\frac{q_{3}\left(W_{b_{3}}-W_{b}\left(t\right)\right)}{V}, \end{array}$$

and

(39) $$W_{a}\left(t\right)+10^{\mathrm{pH}(t)-14}-10^{-\mathrm{pH}(t)}+W_{b}\left(t\right)\frac{1+2\times 10^{\mathrm{pH}\left(t\right)-K_{2}}}{1+10^{K_{1}-\mathrm{pH}\left(t\right)}+10^{\mathrm{pH}\left(t\right)-K_{2}}}=0.$$

State variables $W_{a}$ and $W_{b}$ are reaction invariants. The process manipulated variable is the base NaOH stream denoted as $q_{1}$, while the controlled variable is the pH of the product. The buffer flow rate $q_{2}$ and base flow rate $q_{3}$ remain constant. $W_{a_{1}}$, $W_{a_{2}}$, $W_{a_{3}}$, $W_{b_{1}}$, $W_{b_{2}}$, $W_{b_{3}}$, *V*, $K_{1}$ and $K_{2}$ are constants [19]. The fundamental model given above is utilized only for process simulation, while various Wiener models are used in MPC.

### 4.2. Model Identification and Validation

Two classes of Wiener models are considered: classical and parallel. Neural networks with two layers defined by Equation (5) are utilized in nonlinear static blocks in both models. We use two sets of data generated from the open-loop simulation of the fundamental model for model identification: training and validation data sets. The first set is used only to identify model parameters, while the second set is used to assess model accuracy. All models are found using the same identification procedure. It consists of the following steps:

1. Initialization of the identification procedure. The number of model branches $n_{g}$ ($n_{g}=1$ for the classical Wiener model), the number of hidden nodes in each nonlinear block $K_{1},\ldots ,K_{n_{g}}$, the order of dynamics of linear blocks (defined by integers $n_{A}$ and $n_{B}$), the number of maximal optimization steps used during identification are defined. All model parameters, i.e., parameters of linear dynamical blocks and nonlinear static blocks, are initialized randomly.
2. A nonlinear optimization solver is used to calculate model parameters. The objective of optimization is to minimize the model error for the training data set defined as
  (40) $$E=\sum_{k=1}^{k_{\max}}{\left(y^{\mathrm{mod}}\left(k\right)-y\left(k\right)\right)}^{2},$$
  where $y^{\mathrm{mod}}\left(k\right)$ and y(k) are the model output value and the output value from the training data set, respectively, for the current sampling instant *k*; $k_{\max}$ is the number of available data samples. This work uses the Sequential Quadratic Programming (SQP) solver for nonlinear optimization. Model error for the training data set, denoted by $E_{\mathrm{train}}$, is calculated when optimization is completed.
3. Model error for the validation data set, denoted by $E_{\mathrm{val}}$, is also calculated.
4. Steps 1–4 are repeated a few times, which leads to finding a few models. Of course, initialization of model parameters may have an impact on model accuracy and it may be necessary to repeat identification for the same structure. This is because gradient-based nonlinear optimization is used during identification. Nonlinear optimization may terminate at a shallow local minimum. The finally chosen model has the lowest validation error.

**Figure 3:**
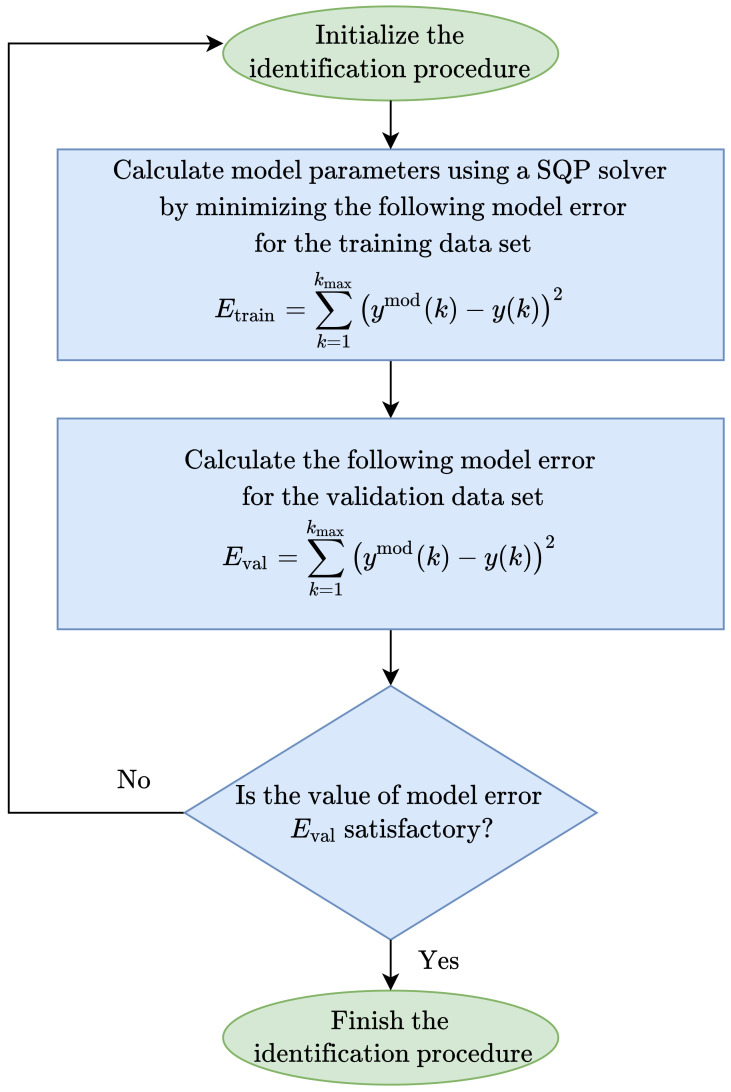
Graphical illustration of the Wiener model identification procedure.

The flowchart of the model identification procedure is presented in Figure 3.

The above identification procedure is independently repeated for different model configurations. This work considers the influence of the number of branches in the parallel model and the number of hidden nodes in neural networks used in nonlinear static blocks. The second order of linear dynamic blocks is always used, i.e., $n_{A}=n_{B}=2$. According to previous research [16,36,38], the second order of dynamics is sufficient for the considered process. Both training and validation data sets used in this work consist of 5000 data samples each.

Many classical and parallel Wiener models have been identified using the abovementioned procedure. We consider the classical Wiener model and parallel ones with two, three and four branches, i.e., $n_{g}=1,\ldots ,4$. In each case, the number of hidden nodes in neural networks varies from one to five, i.e., $K_{g}=1,\ldots ,5$. The activation function of hidden nodes is φ=tanh. Table 1 shows the obtained numerical results of model errors. For each model structure, training and validation errors of the best model are shown, $E_{\mathrm{train}}$ and $E_{\mathrm{val}}$, respectively. Moreover, the percentage relative validation error denoted as $E_{\mathrm{val}}^{\mathrm{relative}}$ is specified. It indicates how the validation error of a particular model compares to that of the best classical Wiener model, i.e., the model with five hidden nodes in the nonlinear block. Such a classical Wiener model has been considered in previous research [16]; using a greater number of nodes is discouraged as they do not lead to model improvement.

Firstly, we compare the results for parallel neural Wiener models with two branches, i.e., $n_{g}=2$. We observe that the model with three hidden nodes, i.e., $K_{g}=3$, results in the lowest relative validation error, equal to 40.29% of that possible for the classical Wiener model. Increasing the number of hidden nodes results in increasing the validation error. Secondly, we compare the results for parallel Wiener models with three branches. We observe that for one hidden node in both branches ($K_{g}=1$), the relative validation error is greater than that for the classical model with five nodes. The best results are again obtained for three hidden nodes with the lowest relative validation error, equal to 39.56%. Increasing the number of hidden nodes increases the number of validation errors. Interestingly, the increase in the number of branches from two to three does not significantly improve model accuracy; both models with three hidden nodes practically have very similar errors. Finally, let us analyze parallel Wiener models with four branches, i.e., $K_{g}=4$. Generally, all obtained models are much worse than the classical Wiener model. The best relative validation error equals 139.81% while the worst one is 3786.40%. For the considered benchmark process, four branches turn out to be unnecessary and badly influence model accuracy. Moreover, such models have multiple parameters and the nonlinear optimization procedure takes more time to find a reasonable solution than in the case of simpler model structures. We also verified parallel Wiener models with five branches and the results are even worse.

**Figure 4:**
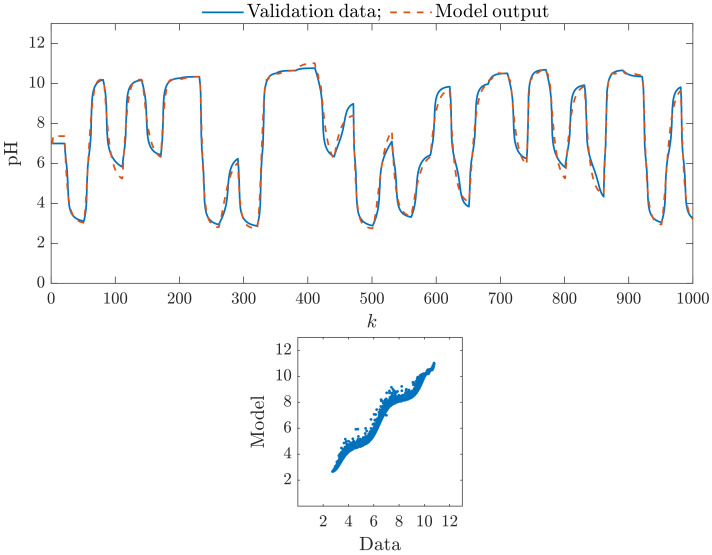
The classical Wiener model with five hidden nodes in the nonlinear static block (K=5): the first 1000 samples of the validation data set vs. the model output (**top**), the relationship between the whole validation data set and the model output (**bottom**).

Let us compare some of the obtained models graphically. It shows how they try to mimic the process represented by the validation data set. Figure 4 presents the results for the classical Wiener model with five hidden nodes in the nonlinear static block (K=5). The top panel compares the first 1000 samples of the validation data set vs. the model output. The bottom panel shows the relationship between the whole validation data set and the model output. In general, we can see that the rudimentary Wiener model is quite precise. Hence, whether and to what extent the parallel structure can increase the model accuracy is interesting.

**Figure 5:**
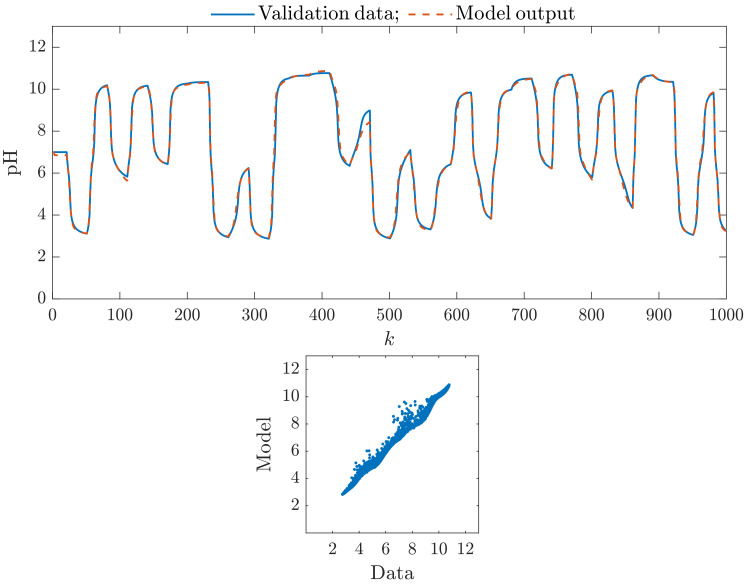
The parallel Wiener model with two branches, each of which has three hidden nodes ($n_{g}=2$, $K_{1}=K_{2}=3$): the first 1000 samples of the validation data set vs. the model output (**top**), the relationship between the whole validation data set and the model output (**bottom**).

**Figure 6:**
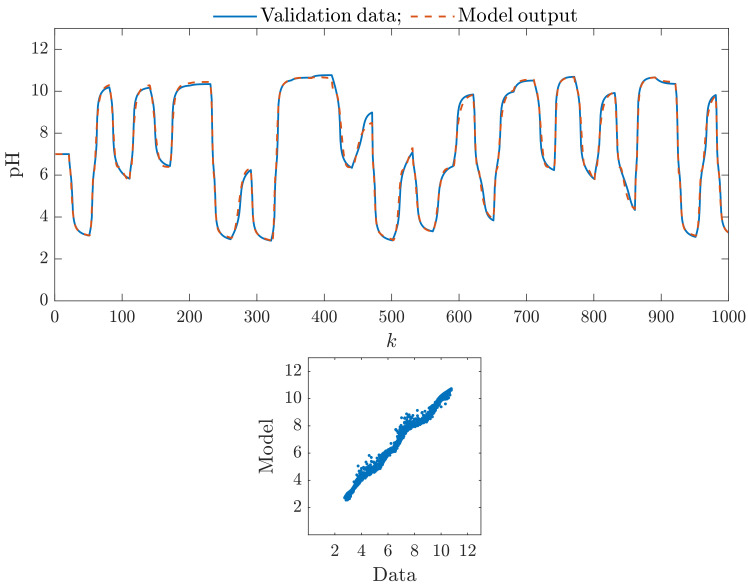
The parallel Wiener model with three branches, each of which has three hidden nodes ($n_{g}=3$, $K_{1}=K_{2}=K_{3}=3$): the first 1000 samples of the validation data set vs. the model output (**top**), the relationship between the whole validation data set and the model output (**bottom**).

**Figure 7:**
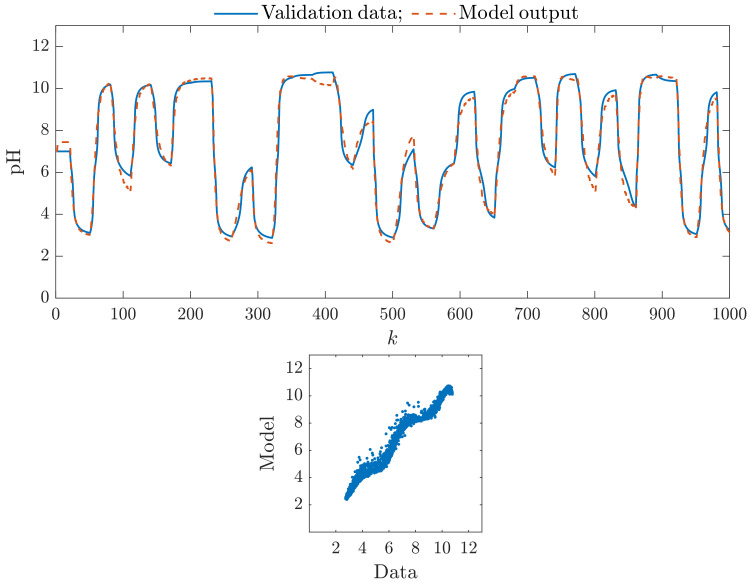
The parallel Wiener model with four branches, each of which has four hidden nodes ($n_{g}=4$, $K_{1}=K_{2}=K_{3}=K_{4}=4$): the first 1000 samples of the validation data set vs. the model output (**top**), the relationship between the whole validation data set and the model output (**bottom**).

**Figure 8:**
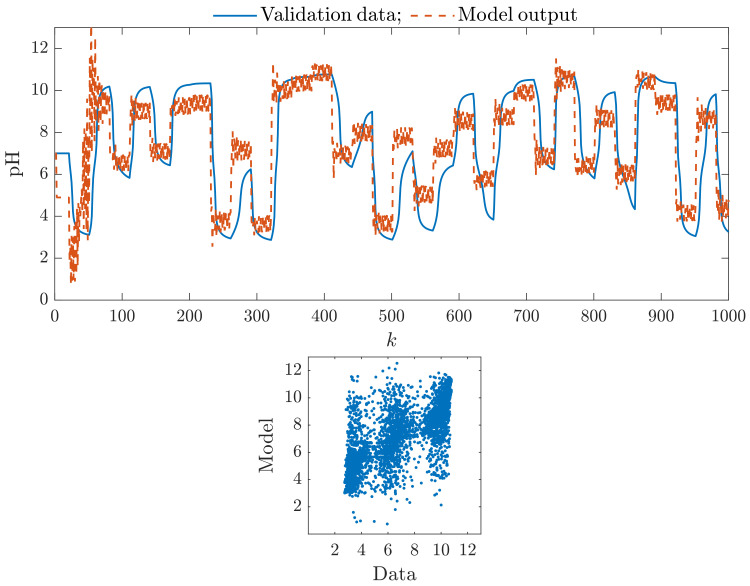
The parallel Wiener model with four branches, each of which has five hidden nodes ($n_{g}=4$, $K_{1}=K_{2}=K_{3}=K_{4}=5$): the first 1000 samples of the validation data set vs. the model output (**top**), the relationship between the whole validation data set and the model output (**bottom**).

Figure 5 shows the efficiency of the parallel Wiener model with two branches, each of which has three hidden nodes ($n_{g}=2$, $K_{1}=K_{2}=3$); Figure 6 shows the efficiency of the parallel Wiener model with three branches, each of which has three hidden nodes ($n_{g}=3$, $K_{1}=K_{2}=K_{3}=3$). We observe that these models have better accuracy than the classical Wiener model. The second one, i.e., the model with three branches, is slightly better. Figure 7 shows the efficiency of the parallel Wiener model with four branches, each of which has four hidden nodes ($n_{g}=4$, $K_{1}=K_{2}=K_{3}=K_{4}=4$). Unfortunately, although the model is the best among all models with four branches, it is noticeably worse than the classical model and parallel models with two and three branches. Finally, Figure 8 shows the efficiency of the parallel Wiener model with four branches, each of which has five hidden nodes ($n_{g}=4$, $K_{1}=K_{2}=K_{3}=K_{4}=5$). In this case, due to overparameterization, the model is very imprecise.

All things considered, parallel Wiener models with two branches make it possible to obtain an error as low as 40% of that observed when the classical Wiener model is used. A slight improvement is provided by models with three parallel branches, while more complex models increase the error due to overparameterization.

### 4.3. Predictive Control of the Neutralization Process

Having found a set of Wiener models and compared them in an open loop, evaluating how they perform in closed-loop MPC control is interesting. In MPC algorithms, we mainly use the classical neural Wiener model with five hidden nodes and the best parallel neural Wiener model with three branches, each of which has three hidden nodes. We also use more complicated models. We consider two MPC algorithms: the discussed MPC-NPLPT algorithm with online linearization and quadratic optimization and the general MPC scheme with Nonlinear Optimization (MPC-NO). The latter uses nonlinear models for prediction, meaning a nonlinear optimization task must be solved at each sampling instant online. We want to obtain the performance of our computationally efficient MPC-NPLPT scheme as close to that of MPC-NO as possible. The following parameters are used in two considered MPC algorithms: N=10, $N_{u}=3$ and λ=0.25 [16].

**Figure 9:**
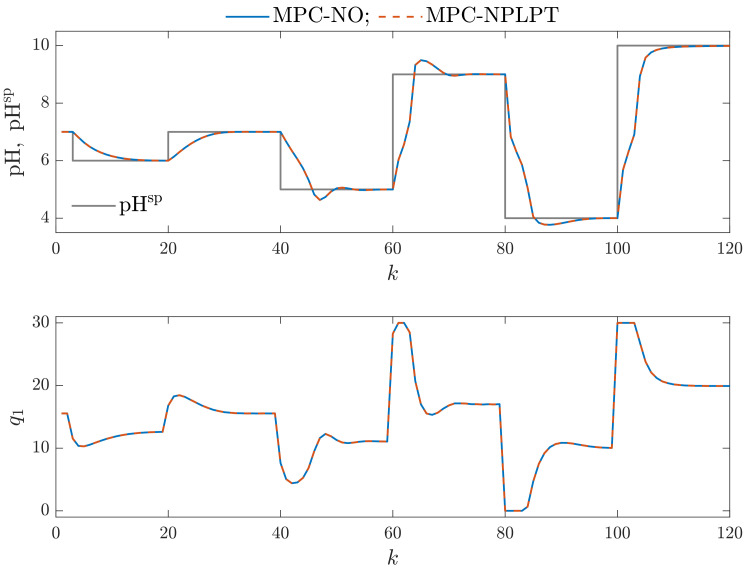
Simulation results: MPC-NO vs. MPC-NPLPT algorithms; both algorithms use the classical Wiener model.

**Figure 10:**
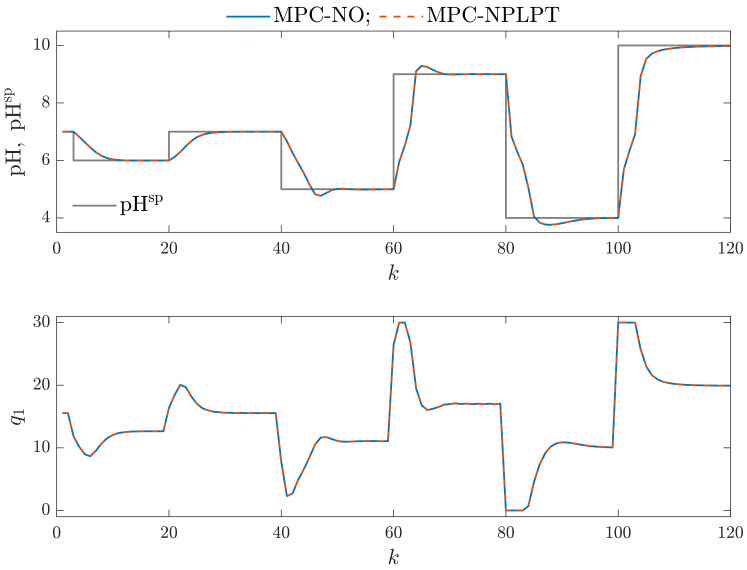
Simulation results: MPC-NO vs. MPC-NPLPT algorithms; both algorithms use the parallel Wiener model with three branches.

**Figure 11:**
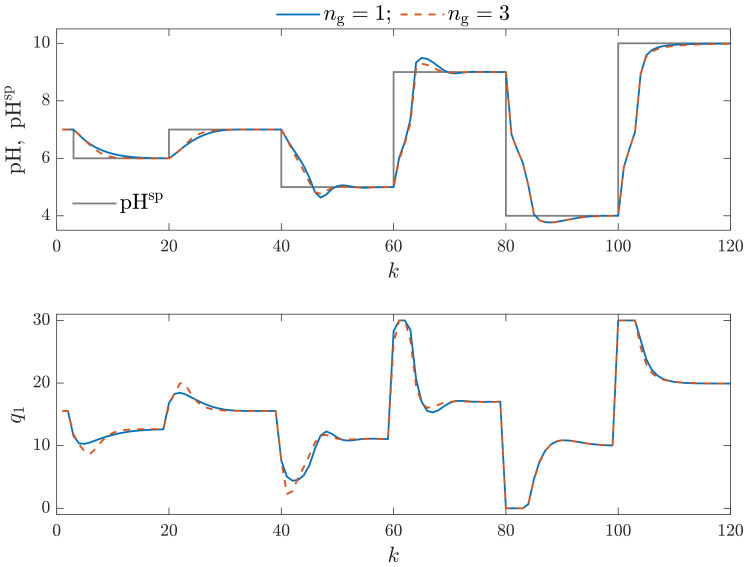
Simulation results: the MPC-NPLPT algorithm using the classical Wiener model vs. the MPC-NPLPT algorithms using the parallel Wiener ($n_{g}=1$) model with three branches ($n_{g}=3$).

This work performs a multicriterial control quality assessment of MPC algorithms. For this purpose, we evaluate the control quality using the following statistical indices: the Mean Squared Error (MSE), the Mean Absolute Error (MAE), the Gauss standard deviation ($\sigma_{G}$), the Huber standard deviation ($\sigma_{H}$), the scale factor of the alpha-stable distribution (γ) and the rational entropy ($H_{R}$). The obtained numerical values of these indicators are presented in Table 2 and the calculation times necessary by MPC algorithms are given in Table 3. We consider MPC-NO and MPC-NPLPT algorithms for classical and the chosen parallel Wiener models. We can formulate the following observations:

1. The control quality indicators obtained for the MPC-NPLPT algorithm are practically the same as those for the MPC-NO control method. That means that our control algorithm is very efficient. Advanced online trajectory linearization makes it possible to use simple quadratic optimization; nonlinear programming is unnecessary. This observation can also be verified when we consider process time trajectories. Figure 9 compares simulation results of MPC-NO and MPC-NPLPT algorithms; both of them use the classical Wiener model. The controlled variable and the setpoint trajectory are displayed in the top panel. The manipulated variable is shown in the bottom panel. Although they use a completely different computational scheme, we can see that both algorithms’ trajectories are very close. The same observations can be noted from Figure 10, which compares simulation results of MPC-NO and MPC-NPLPT algorithms, but now both algorithms use the parallel Wiener model with three branches.
2. From Table 2, we can find out that better control quality is achieved when MPC algorithms use the parallel Wiener model rather than the classical structure. The following indices are significantly reduced when the parallel model is used: MAE, $\sigma_{H}$, γ and rational entropy ($H_{R}$). The rest of the indices (MSE and $\sigma_{G}$) are slightly lower. Figure 11 presents the obtained trajectories possible when the same control algorithm MPC-NPLPT is used, but classical and parallel Wiener models are used for prediction. We can clearly see that the parallel model control scheme offers better control quality. Namely, the settling time is shorter and the overshoot is smaller.
3. Of course, increasing the number of model branches is likely to increase the computation time. Therefore, Wiener models with as few branches as possible should be used. Table 3 details calculation times of studied MPC algorithms for classical and parallel Wiener models. As all simulations are performed in MATLAB (not in a real industrial control system), we are interested in a relative comparison between the studied algorithms. Hence, all results are scaled so that the calculation time for the computationally demanding MPC-NO algorithm based on the classical Wiener model is assumed to be equal to 100%. It is interesting to note that increasing the number of branches significantly influences the calculation time of the MPC-NO algorithm with nonlinear optimization. On the other hand, the time required by the MPC-NPLPT algorithm developed and recommended in our work is significantly shorter and not influenced by the number of model branches. It is because the MPC-NPLPT quadratic optimization problem has a predominant influence on calculation time.

**Figure 12:**
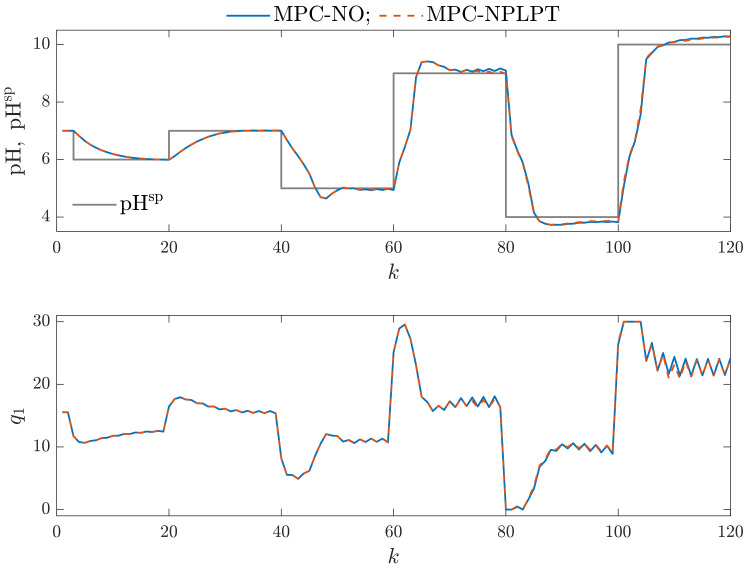
Simulation results: MPC-NO vs. MPC-NPLPT algorithms; both algorithms use the parallel Wiener model with $n_{g}=4$ branches and neural networks with $K_{g}=4$ hidden nodes.

**Figure 13:**
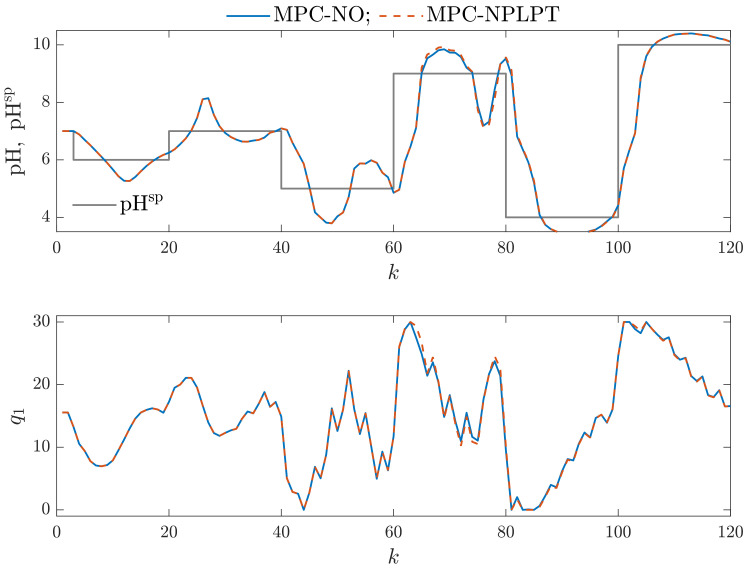
Simulation results: MPC-NO vs. MPC-NPLPT algorithms; both algorithms use the parallel Wiener model with $n_{g}=4$ branches and neural networks with $K_{g}=5$ hidden nodes.

It is interesting whether more complicated parallel Wiener models may be used in MPC. From Table 1 and Figure 7 and Figure 8, we can see that increasing the number of model branches does not lead to improving open-loop model accuracy. As far as closed-loop model performance is concerned, let us consider Figure 12, which shows simulation results of MPC-NO and MPC-NPLPT algorithms that use the parallel Wiener model with $n_{g}=4$ branches and neural networks with $K_{g}=4$ hidden nodes. This is the best model among all models with four branches. Both algorithms produce the same trajectories, which is good because it means that our MPC-NPLPT algorithm perfectly mimics the computationally demanding MPC-NO method. Unfortunately, the control quality is generally much worse than in the case of parallel Wiener models with three branches. The manipulated variable has an oscillatory behavior, resulting in the controlled variable oscillating. Such an unwanted phenomenon occurs when the controlled variable value is close to the current set point value. Similarly, Figure 13 compares the same MPC algorithms, but now both algorithms use the parallel Wiener model with $n_{g}=4$ branches and neural networks with $K_{g}=5$ hidden nodes. This is the worst model among all models with four branches. The control results are very bad. The controlled variable of the process practically does not stabilize on the required setpoint. There are frequent oscillations of manipulated and controlled variables. The amplitude of the oscillations is significant, and as a result, large overshoots are obtained.

## 5. Conclusions

This work is concerned with parallel Wiener models. Firstly, it details a computationally efficient MPC algorithm for the parallel Wiener model. The idea is to avoid nonlinear prediction and nonlinear online optimization. Conversely, an online linear approximation of the process predicted trajectory is successively computed, leading to a relatively simple quadratic optimization. Secondly, parallel Wiener models are compared with classical ones for a benchmark neutralization process. Model accuracy is compared in the open-loop configuration. We find out that the parallel Wiener models really offer significantly better accuracy than the classical model. It is also necessary to stress that excessively complicated parallel models, with too many branches, suffer from overparameterization and cannot be trained fast. Hence, we suggest using parallel Wiener models with only a few branches for the considered process. Thirdly, parallel Wiener models are verified in MPC. Of note, the discussed MPC algorithm with online linearization and fast quadratic programming for the neutralization system produces practically the same results as the rudimentary MPC method with a fully nonlinear approach. Interestingly, control quality based on MPC algorithms based on parallel Wiener models is better than the classical model. However, the gain of using more complex models is not very significant due to the closed-loop negative feedback mechanism present in MPC.

## Figures and Tables

**Table 1:** Training and validation errors of classical and parallel Wiener models.

Model Type	$n_{g}$	$K_{g}$	$E_{\mathrm{train}}$	$E_{\mathrm{val}}$	$E_{\mathrm{val}}^{\mathrm{relative}}$
Classical neural Wiener	1	1	$5.01\times 10^{2}$	$5.03\times 10^{2}$	122.08%
	1	2	$4.97\times 10^{2}$	$4.99\times 10^{2}$	121.11%
	1	3	$4.81\times 10^{2}$	$4.84\times 10^{2}$	117.47%
	1	4	$4.37\times 10^{2}$	$4.42\times 10^{2}$	107.28%
	1	5	$4.07\times 10^{2}$	$4.12\times 10^{2}$	100.00%
Parallel neural Wiener	2	1	$2.28\times 10^{2}$	$2.61\times 10^{2}$	0063.34%
	2	2	$1.39\times 10^{2}$	$1.88\times 10^{2}$	0045.63%
	2	3	$1.20\times 10^{2}$	$1.66\times 10^{2}$	0040.29%
	2	4	$1.35\times 10^{2}$	$1.81\times 10^{2}$	0043.93%
	2	5	$1.84\times 10^{2}$	$2.24\times 10^{2}$	0054.36%
	3	1	$4.81\times 10^{2}$	$4.80\times 10^{2}$	116.50%
	3	2	$1.56\times 10^{2}$	$1.91\times 10^{2}$	0046.35%
	3	3	$1.41\times 10^{2}$	$1.63\times 10^{2}$	0039.56%
	3	4	$1.34\times 10^{2}$	$1.75\times 10^{2}$	0042.47%
	3	5	$1.47\times 10^{2}$	$1.87\times 10^{2}$	0045.38%
	4	1	$7.36\times 10^{3}$	$6.73\times 10^{3}$	1633.50%
	4	2	$1.64\times 10^{3}$	$1.65\times 10^{3}$	400.49%
	4	3	$6.19\times 10^{2}$	$6.57\times 10^{2}$	159.47%
	4	4	$5.78\times 10^{2}$	$5.76\times 10^{2}$	139.81%
	4	5	$1.53\times 10^{4}$	$1.56\times 10^{4}$	3786.40%

**Table 2:** Multi-criteria control quality indicators of MPC algorithms with classical and parallel Wiener models.

Model Type	MPC Algorithm	MSE	MAE	$\sigma_{G}$	$\sigma_{H}$	γ	$H_{R}$
Classical neural Wiener	MPC-NO	$1.4375\times 10^{0}$	$5.3123\times 10^{-1}$	$1.2007\times 10^{0}$	$1.1652\times 10^{-1}$	$4.9899\times 10^{-2}$	$4.4580\times 10^{-1}$
	MPC-NPLPT	$1.4375\times 10^{0}$	$5.3123\times 10^{-1}$	$1.2007\times 10^{0}$	$1.1649\times 10^{-1}$	$4.9887\times 10^{-2}$	$4.4580\times 10^{-1}$
Parallel neural Wiener, $n_{g}=3$	MPC-NO	$1.4329\times 10^{0}$	$5.0995\times 10^{-1}$	$1.1971\times 10^{0}$	$7.8351\times 10^{-2}$	$3.7433\times 10^{-2}$	$4.1628\times 10^{-1}$
	MPC-NPLPT	$1.4332\times 10^{0}$	$5.0992\times 10^{-1}$	$1.1972\times 10^{0}$	$7.8945\times 10^{-2}$	$3.7596\times 10^{-2}$	$4.1060\times 10^{-1}$

**Table 3:** Calculation times for MPC algorithms with classical and parallel Wiener models.

Model Type	MPC Algorithm	Time
Classical neural Wiener	MPC-NO	100.00%
	MPC-NPLPT	142.01%
Parallel neural Wiener, $n_{g}=3$	MPC-NO	146.01%
	MPC-NPLPT	156.01%

## Data Availability

The data presented in this study are available on request from the corresponding author.
